# Efficacy of Trametinib in Alleviating Cisplatin-Induced Acute Kidney Injury: Inhibition of Inflammation, Oxidative Stress, and Tubular Cell Death in a Mouse Model

**DOI:** 10.3390/molecules29122881

**Published:** 2024-06-17

**Authors:** Joung Eun Lee, Jung-Yeon Kim, Jaechan Leem

**Affiliations:** 1 Department of Emergency Medicine, School of Medicine, Daegu Catholic University, Daegu 42472, Republic of Korea; zaiaz12815@daum.net; 2 Department of Immunology, School of Medicine, Daegu Catholic University, Daegu 42472, Republic of Korea; jy1118@cu.ac.kr

**Keywords:** trametinib, cisplatin, acute kidney injury, inflammation, oxidative stress, apoptosis, necroptosis

## Abstract

Cisplatin, a platinum-based chemotherapeutic, is effective against various solid tumors, but its use is often limited by its nephrotoxic effects. This study evaluated the protective effects of trametinib, an FDA-approved selective inhibitor of mitogen-activated protein kinase kinase 1/2 (MEK1/2), against cisplatin-induced acute kidney injury (AKI) in mice. The experimental design included four groups, control, trametinib, cisplatin, and a combination of cisplatin and trametinib, each consisting of eight mice. Cisplatin was administered intraperitoneally at a dose of 20 mg/kg to induce kidney injury, while trametinib was administered via oral gavage at 3 mg/kg daily for three days. Assessments were conducted 72 h after cisplatin administration. Our results demonstrate that trametinib significantly reduces the phosphorylation of MEK1/2 and extracellular signal-regulated kinase 1/2 (ERK1/2), mitigated renal dysfunction, and ameliorated histopathological abnormalities. Additionally, trametinib significantly decreased macrophage infiltration and the expression of pro-inflammatory cytokines in the kidneys. It also lowered lipid peroxidation by-products, restored the reduced glutathione/oxidized glutathione ratio, and downregulated NADPH oxidase 4. Furthermore, trametinib significantly inhibited both apoptosis and necroptosis in the kidneys. In conclusion, our data underscore the potential of trametinib as a therapeutic agent for cisplatin-induced AKI, highlighting its role in reducing inflammation, oxidative stress, and tubular cell death.

## 1. Introduction

Cisplatin, a platinum-containing chemotherapeutic agent, is extensively used for treating various solid tumors [1]. Despite its effectiveness in cancer therapy, a significant drawback of cisplatin is its nephrotoxicity, which can manifest as acute kidney injury (AKI) and potentially lead to long-term renal impairment [2]. The development of therapeutic strategies to mitigate cisplatin-induced nephrotoxicity is crucial, as it limits the optimal dosage and duration of treatment, thus impacting the overall effectiveness of the therapy.

Cisplatin-induced nephrotoxicity is primarily driven by inflammation, oxidative stress, and tubular cell death [2,3,4]. The inflammatory process is initiated when cisplatin induces the release of cytokines and damage-associated molecular patterns, attracting immune cells, like macrophages, to the kidneys. These cells exacerbate damage through the release of pro-inflammatory mediators. Additionally, the entry of cisplatin into renal cells and its subsequent metabolic reactions generate reactive oxygen species (ROS), leading to significant oxidative damage and exacerbating kidney injury [5]. This oxidative stress is a critical driver of the apoptosis observed in tubular epithelial cells, characterized by mitochondrial dysfunction and the activation of cellular death pathways [6,7]. Recent studies also highlight the involvement of alternative cell death mechanisms, such as necroptosis, ferroptosis, and pyroptosis, in the progression of nephrotoxicity, further disrupting renal function and integrity [8,9,10].

The extracellular signal-regulated kinase (ERK) pathway is one of the key signaling cascades in the mitogen-activated protein kinase (MAPK) family [11]. The activation of the ERK pathway typically begins at the cell surface with the binding of growth factors to receptor tyrosine kinases. This binding initiates a cascade that includes the phosphorylation of MAP kinase kinase 1/2 (MEK1/2) and subsequent activation of ERK1/2 [11]. This pathway is involved in various cellular processes, including inflammation, oxidative stress, and cell death [11,12,13], and has been implicated in the modulation of cisplatin-induced nephrotoxicity [14,15,16]. Notably, the ERK pathway inhibitor U0126 has shown protective effects against cisplatin-induced AKI by reducing inflammation and apoptosis [14,15,16].

Drug repositioning involves identifying new therapeutic uses for existing drugs, significantly reducing development costs and time [17]. Trametinib, a potent and selective MEK1/2 inhibitor, was approved by the Food and Drug Administration (FDA) in 2013 following the METRIC study, which demonstrated its efficacy in treating advanced or metastatic melanomas with specific B-Raf proto-oncogene (BRAF) mutations [18]. Its biological action involves inhibiting MEK1/2, preventing the activation of ERK1/2, and thereby disrupting downstream signaling pathways crucial for cell proliferation and survival [18]. Clinically, trametinib has been extensively studied in combination with other targeted therapies, such as dabrafenib, to enhance its efficacy in treating BRAF-mutant melanoma [19]. These studies have demonstrated improved overall survival and progression-free survival in patients. However, trametinib is associated with several side effects, including rash, diarrhea, and lymphedema, as well as more serious adverse events like cardiomyopathy and retinal vein occlusion [19].

We believe that trametinib is a potential candidate to inhibit the pathological processes associated with cisplatin-induced kidney injury due to its ability to attenuate inflammation, oxidative stress, and cell death by interfering with the MEK/ERK pathway. Although a recent animal study reported that trametinib is ineffective against cisplatin-induced nephrotoxicity [20], our findings contradict these results. The present study aims to evaluate the potential of trametinib to mitigate these pathological processes in a mouse model of cisplatin-induced AKI. Specifically, we will perform functional and histopathological evaluations of the kidneys, and assess macrophage infiltration, cytokine production, oxidative stress markers, and the pathways of apoptosis and necroptosis. These assessments are expected to provide valuable insights into the potential repositioning of trametinib for managing cisplatin-induced nephrotoxicity.

## 2. Results

### 2.1. Effects of Trametinib on Cisplatin-Induced Renal Dysfunction and Structural Damage

**Figure 1:**
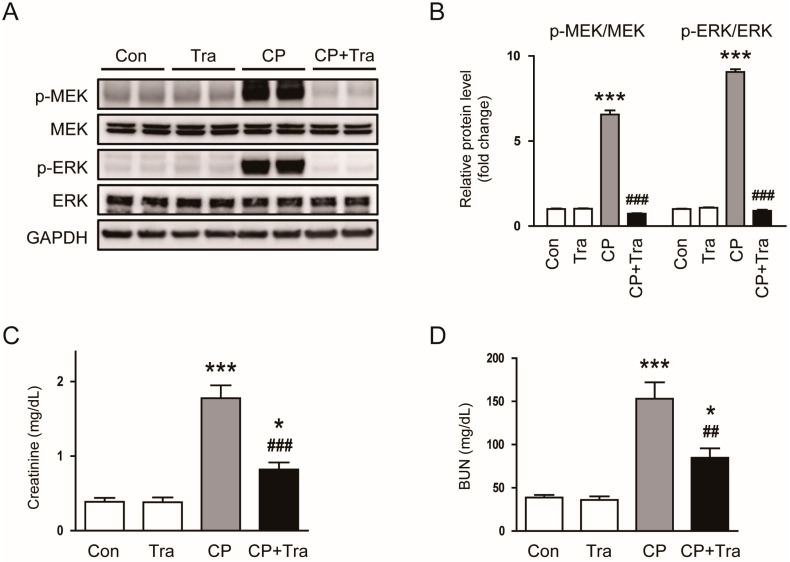
Effects of trametinib on the MEK-ERK pathway and renal function in cisplatin-injected mice. (**A**) Western blotting of p-MEK and p-ERK in kidney tissues. (**B**) Bar graphs showing the quantified band densities of p-MEK and p-ERK, normalized to the total protein levels (n = 6 per group). (**C**) Serum creatinine levels (n = 8 per group). (**D**) BUN levels (n = 8 per group). The data are presented as mean ± SEM. * *p* < 0.05 and *** *p* < 0.001 vs. Con. ^##^ *p* < 0.01 and ^###^ *p* < 0.001 vs. CP.

Initially, we evaluated the phosphorylation of MEK1/2 and ERK1/2 in all groups. Phosphorylation of MEK1/2 and ERK1/2 largely increased in kidney tissues 72 h post-administration of cisplatin (Figure 1A,B). Trametinib treatment completely suppressed the phosphorylation of MEK1/2 and ERK1/2 in cisplatin-injected mice (p-MEK1/2: 6.54 ± 0.24 versus 0.72 ± 0.04, *p* < 0.001; p-ERK1/2: 9.07 ± 0.16 versus 0.91 ± 0.06, *p* < 0.001; Figure 1A,B). Cisplatin injection resulted in significant increases in serum creatinine and blood urea nitrogen (BUN) levels compared to control mice (Figure 1C,D). In contrast, trametinib treatment significantly mitigated the cisplatin-induced elevations in serum creatinine and BUN levels (creatinine: 1.78 ± 0.22 mg/dL versus 0.79 ± 0.12, *p* < 0.001; BUN: 153.1 ± 19.0 mg/dL versus 84.8 ± 10.8, *p* < 0.01; Figure 1C,D). Additionally, treatment with trametinib alone did not result in significant differences in serum creatinine and BUN levels compared to the control mice (Figure 1C,D).

**Figure 2:**
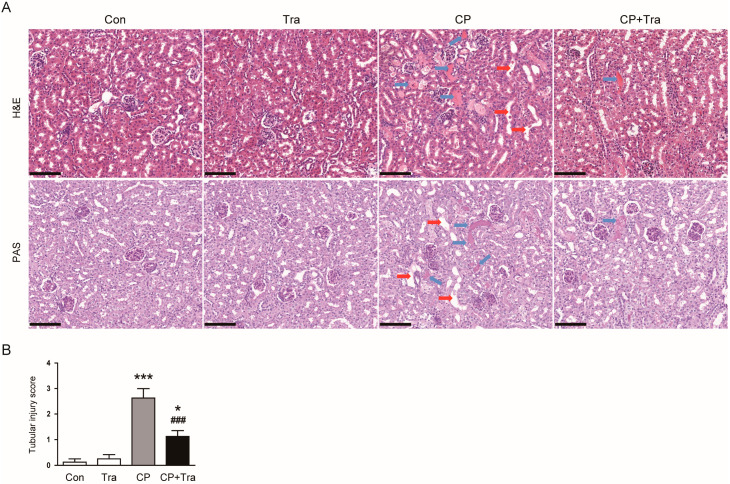
Effects of trametinib on histopathological abnormalities in cisplatin-injected mice. (**A**) Representative images of H&E or PAS-stained kidney sections. Scale bar = 100 μm. Red arrows point to tubular dilatation, and blue arrows to cast deposition in the tubular lumens. (**B**) Tubular injury scores (n = 8 per group). The data are presented as mean ± SEM. * *p* < 0.05 and *** *p* < 0.001 versus Con. ^###^ *p* < 0.001 versus CP.

Employing hematoxylin and eosin (H&E) and periodic acid-Schiff (PAS) staining techniques, we observed significant tubular damage in the kidneys of mice subjected to cisplatin injection, characterized by notable tubular dilatation and the presence of casts (Figure 2A,B). Intriguingly, treatment with trametinib resulted in a marked amelioration of these histopathological changes, which was quantitatively reflected in the substantially lower scores for tubular injury (2.63 ± 0.38 versus 1.13 ± 0.23, *p* < 0.001; Figure 2A,B).

**Figure 3:**
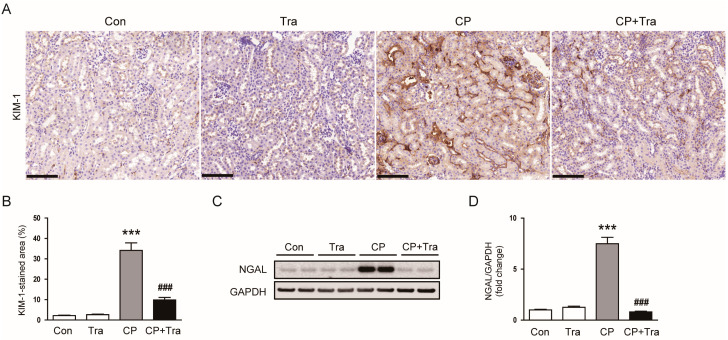
Effects of trametinib on expression of tubular injury markers, KIM-1 and NGAL, in cisplatin-injected mice. (**A**) Representative images of IHC staining for KIM-1. Scale bar = 100 μm. (**B**) Quantitative analysis of KIM-1 positive staining (n = 8 per group). (**C**) Western blot results for NGAL in kidney tissues. (**D**) Bar graph depicting the quantified band densities of NGAL, normalized against GAPDH levels (n = 6 per group). The data are presented as mean ± SEM. *** *p* < 0.001 versus Con. ^###^ *p* < 0.001 versus CP.

Our immunohistochemistry (IHC) staining indicated heightened expression of kidney injury molecule-1 (KIM-1) in mice injected with cisplatin (Figure 3A,B). Notably, trametinib treatment resulted in a significant reduction in KIM-1 expression, as evidenced by the reduced proportion of the area stained positively for KIM-1 (34.1 ± 3.7% versus 9.8 ± 1.3%, *p* < 0.001; Figure 3A,B). Furthermore, Western blot analysis corroborated these findings, showing a substantial reduction in neutrophil gelatinase-associated lipocalin (NGAL) protein levels in cisplatin-injected mice upon trametinib treatment (7.52 ± 0.63 versus 0.81 ± 0.08, *p* < 0.001; Figure 3C,D).

**Figure 4:**
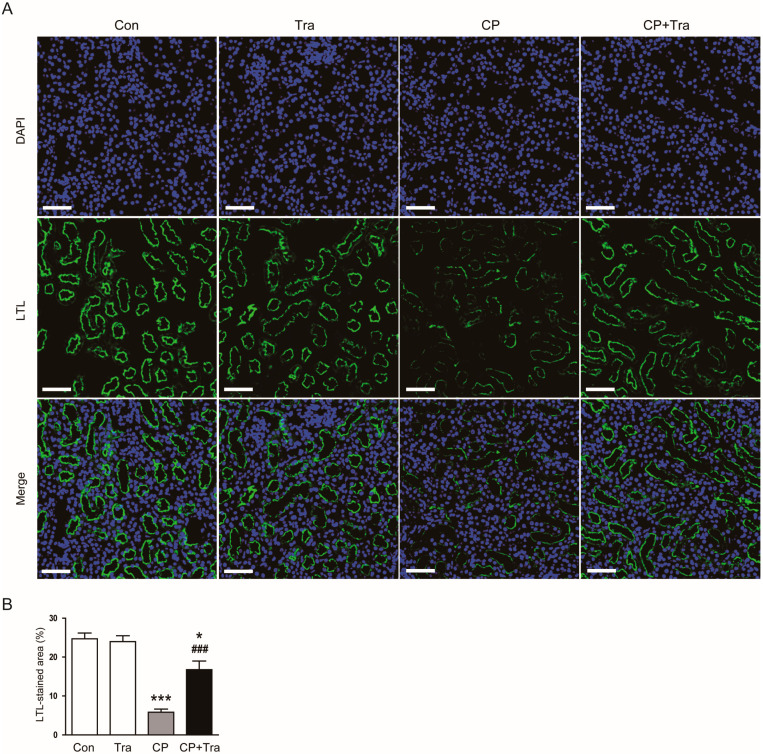
Effects of trametinib on brush border loss of proximal tubules in cisplatin-injected mice. (**A**) IF staining for LTL (green). Nuclei were counterstained with DAPI (blue). Scale bar = 50 μm. (**B**) Quantitative analysis of LTL positive staining (n = 8 per group). The data are presented as mean ± SEM. * *p* < 0.05 and *** *p* < 0.001 versus Con. ^###^ *p* < 0.001 versus CP.

Immunofluorescence (IF) staining on kidney sections using fluorescein isothiocyanate (FITC)-conjugated lotus tetragonolobus lectin (LTL) showed cisplatin injection markedly diminished the LTL-stained area (Figure 4A,B). However, trametinib treatment resulted in a significant increase in the area with positive LTL staining in mice injected with cisplatin (5.9 ± 0.8% versus 16.8 ± 2.2%, *p* < 0.001; Figure 4A,B), indicating an effective preservation of the brush border structure.

### 2.2. Effects of Trametinib on Cisplatin-Induced Inflammation

**Figure 5:**
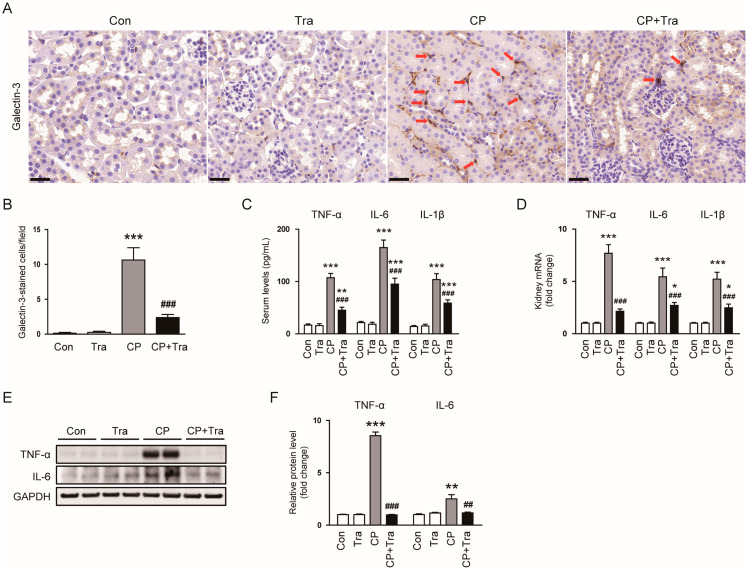
Effects of trametinib on macrophage infiltration and cytokine production in cisplatin-injected mice. (**A**) Representative images of IHC staining for galectin-3. Red arrows indicate positively stained cells. Scale bar = 40 μm. (**B**) Quantitative analysis of galectin-3 positive staining (n = 8 per group). (**C**) Serum levels of TNF-α, IL-6, and IL-1β (n = 8 per group). (**D**) Renal mRNA levels of TNF-α, IL-6, and IL-1β (n = 8 per group). (**E**) Western blot results for TNF-α and IL-6 in kidney tissues. (**F**) Bar graph depicting the quantified band densities of TNF-α and IL-6, normalized against GAPDH levels (n = 6 per group). The data are presented as mean ± SEM. * *p* < 0.05, ** *p* < 0.01, and *** *p* < 0.001 versus Con. ^##^ *p* < 0.01 and ^###^ *p* < 0.001 versus CP.

To understand trametinib’s protective effects against cisplatin-induced AKI, we focused on its influence on the inflammatory responses elicited by cisplatin. Our IHC staining for galectin-3 revealed that cisplatin injection greatly increased macrophage numbers, but trametinib treatment effectively reduced macrophage accumulation in cisplatin-injected mice (10.6 ± 1.7 versus 2.4 ± 0.5, *p* < 0.001; Figure 5A,B). Additionally, trametinib treatment lowered the serum levels of tumor necrosis factor-α (TNF-α), interleukin-6 (IL-6), and IL-1β in cisplatin-injected mice (TNF-α: 107.3 ± 8.2 pg/mL versus 45.1 ± 5.6 pg/mL, *p* < 0.001; IL-6: 164.8 ± 14.4 pg/mL versus 95.1 ± 11.2 pg/mL, *p* < 0.001; IL-1β: 103.8 ± 11.3 pg/mL versus 58.6 ± 6.5 pg/mL, *p* < 0.001; Figure 5C), and also decreased renal mRNA levels of these cytokines (TNF-α: 7.7 ± 0.8 versus 2.1 ± 0.2, *p* < 0.001; IL-6: 5.5 ± 0.8 versus 2.7 ± 0.3, *p* < 0.001; IL-1β: 5.2 ± 0.7 versus 2.5 ± 0.4, *p* < 0.001; Figure 5D). The ability of trametinib to inhibit the expression of TNF-α and IL-6 was further confirmed by Western blot analysis (TNF-α: 8.6 ± 0.4 versus 0.98 ± 0.06, *p* < 0.001; IL-6, 2.5 ± 0.4 versus 1.18 ± 0.07, *p* < 0.01; Figure 5E,F).

**Figure 6:**
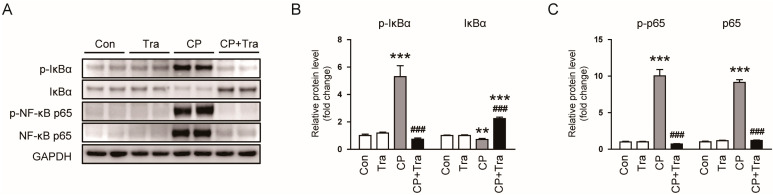
Effects of trametinib on NF-κB activation in cisplatin-injected mice. (**A**) Western blot results for p-IκBα, IκBα, p-NF-κB p65, and NF-κB p65 in kidney tissues. (**B**) Bar graphs depicting the quantified band densities of p-IκBα and IκBα, normalized against GAPDH levels (n = 6 per group). (**C**) Bar graphs depicting the quantified band densities of p-NF-κB p65 and NF-κB p65, normalized against GAPDH levels (n = 6 per group). The data are presented as mean ± SEM. ** *p* < 0.01 and *** *p* < 0.001 versus Con. ^###^ *p* < 0.001 versus CP.

We next examined the effect of trametinib on NF-κB activation in cisplatin-induced AKI. Using Western blot analysis, we found that cisplatin injection elevated levels of p-IκBα, p-NF-κB p65, and NF-κB p65, while reducing IκBα levels (Figure 6A–C). However, these effects induced by cisplatin were significantly reversed when the mice were treated with trametinib (p-IκBα: 5.3 ± 0.8 versus 0.73 ± 0.09, *p* < 0.001; IκBα: 0.72 ± 0.08 versus 2.24 ± 0.12, *p* < 0.001; p-NF-κB p65: 10.1 ± 0.9 versus 0.72 ± 0.06, *p* < 0.001; NF-κB p65: 9.2 ± 0.4 versus 1.12 ± 0.06, *p* < 0.001; Figure 6A–C), indicating its inhibitory effect on the activation of the NF-κB pathway induced by cisplatin.

### 2.3. Effects of Trametinib on Cisplatin-Induced Oxidative Stress

**Figure 7:**
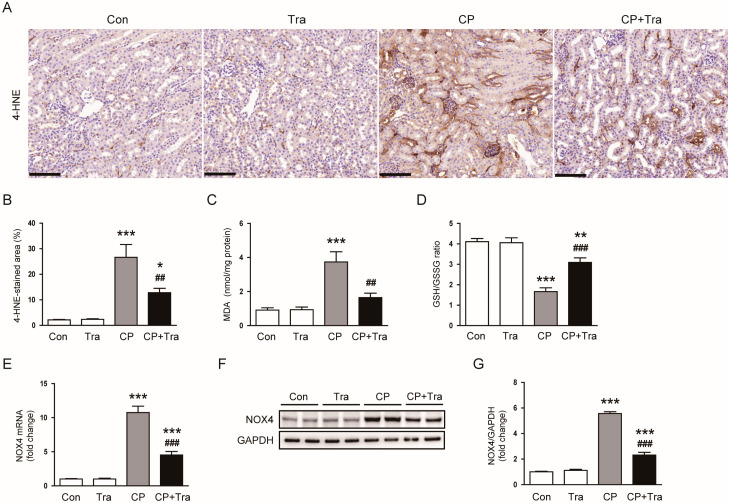
Effects of trametinib on oxidative stress in cisplatin-injected mice. (**A**) Representative images of IHC staining for 4-HNE. Scale bar = 100 μm. (**B**) Quantitative analysis of 4-HNE positive staining (n = 8 per group). (**C**) Renal MDA levels (n = 8 per group). (**D**) The GSH/GSSG ratio (n = 8 per group). (**E**) Renal NOX4 mRNA levels (n = 8 per group). (**F**) Western blot results for NOX4 in kidney tissues. (**G**) Bar graph depicting the quantified band densities of NOX4, normalized against GAPDH levels (n = 6 per group). The data are presented as mean ± SEM. * *p* < 0.05, ** *p* < 0.01, and *** *p* < 0.001 versus Con. ^##^ *p* < 0.01 and ^###^ *p* < 0.001 versus CP.

IHC staining showed that the increase in the area positively stained for 4-HNE was significantly reduced after trametinib treatment (26.6 ± 5.0% versus 12.8 ± 1.7%, *p* < 0.01; Figure 7A,B). Biochemical measurement of malondialdehyde (MDA) levels also demonstrated trametinib’s inhibitory effect on lipid peroxidation in cisplatin-injected mice (3.7 ± 0.6 nmol/mg protein versus 1.7 ± 0.3 nmol/mg protein, *p* < 0.01; Figure 7C). Additionally, the reduction in the reduced glutathione/oxidized glutathione (GSH/GSSG) ratio following cisplatin injection was significantly reversed by trametinib treatment (1.7 ± 0.2 versus 3.1 ± 0.2, *p* < 0.001; Figure 7D). Cisplatin injection also markedly increased the mRNA and protein levels of NADPH oxidase 4 (NOX4) in the kidney (Figure 7E–G). However, trametinib treatment significantly reduced NOX4 expression in cisplatin-injected mice (NOX4 mRNA: 10.7 ± 0.9 versus 4.5 ± 0.5, *p* < 0.001; NOX4 protein: 5.6 ± 0.1 versus 2.3 ± 0.2, *p* < 0.001; Figure 7E–G). These findings collectively suggest that trametinib’s inhibition of the MEK-ERK pathway results in the downregulation of NOX4 and a consequent reduction in oxidative stress.

### 2.4. Effects of Trametinib on Cisplatin-Induced Cell Death

**Figure 8:**
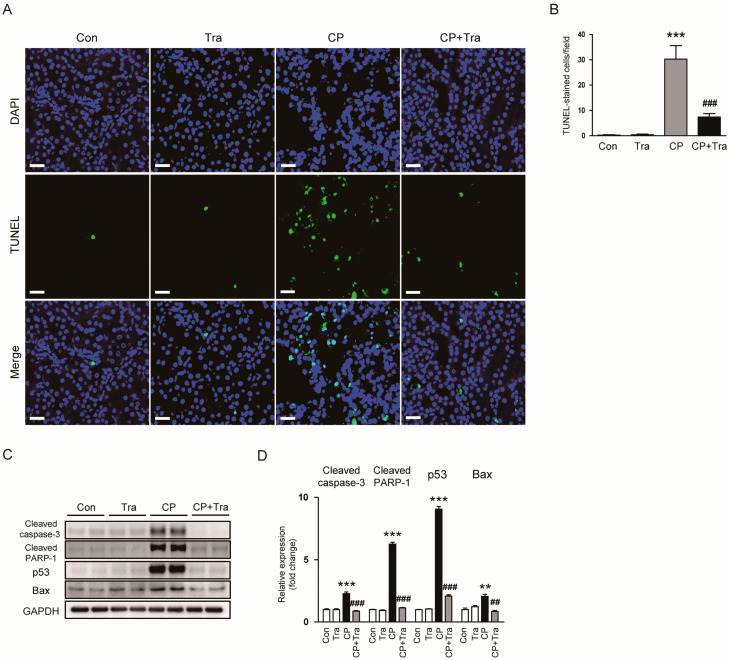
Effects of trametinib on apoptotic cell death in cisplatin-injected mice. (**A**) TUNEL staining (green) on kidney sections. Scale bar = 20 μm. Nuclei were counterstained with DAPI (blue). (**B**) Number of TUNEL-stained cells per field (n = 8 per group). (**C**) Western blot results for cleaved caspase-3, cleaved PARP-1, p53, and Bax in kidney tissues. (**D**) Bar graph depicting the quantified band densities of cleaved caspase-3, cleaved PARP-1, p53, and Bax, normalized against GAPDH levels (n = 6 per group). The data are presented as mean ± SEM. ** *p* < 0.01 and *** *p* < 0.001 versus Con. ^##^ *p* < 0.01 and ^###^ *p* < 0.001 versus CP.

We employed the TdT-mediated dUTP nick end labeling (TUNEL) method to evaluate the influence of trametinib on tubular cell apoptosis following cisplatin injection. Notably, cisplatin injection resulted in a pronounced increase in TUNEL-positive cells within the renal tissue (Figure 8A,B). Contrastingly, trametinib treatment significantly mitigated this effect, leading to a considerable decrease in the count of TUNEL-stained apoptotic cells in mice injected with cisplatin (30.3 ± 5.4 versus 7.4 ± 1.4, *p* < 0.001; Figure 8A,B). Complementing these findings, Western blot analysis further elucidated trametinib’s role, showcasing its capacity to lower the expression of several key proteins associated with apoptosis, such as cleaved caspase-3, cleaved poly(ADP-ribose)-polymerase-1 (PARP-1), p53, and Bax, in the kidneys of cisplatin-injected mice (cleaved caspase-3: 2.29 ± 0.13 versus 0.88 ± 0.05, *p* < 0.001; cleaved PARP-1: 6.25 ± 0.15 versus 1.15 ± 0.04, *p* < 0.001; p53: 9.05 ± 0.21 versus 2.12 ± 0.11, *p* < 0.001; Bax: 2.06 ± 0.18 versus 0.85 ± 0.09, *p* < 0.01; Figure 8C,D).

**Figure 9:**
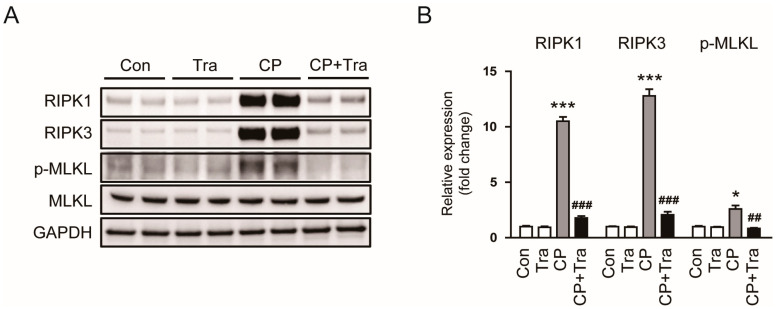
Effects of trametinib on necroptotic cell death in cisplatin-injected mice. (**A**) Western blot results for RIPK1, RIPK3, p-MLKL, and MLKL. (**B**) Quantification of western blots for RIPK1, RIPK3, and p-MLKL (n = 6 per group). GAPDH was used as an internal control. The data are presented as mean ± SEM. * *p* < 0.05 and *** *p* < 0.001 versus Con. ^##^ *p* < 0.01 and ^###^ *p* < 0.001 versus CP.

To understand the impact of trametinib on necroptosis, we examined the effect of trametinib on the receptor-interacting serine/threonine protein kinase 1 (RIPK1)-RIPK3-mixed lineage kinase domain-like protein (MLKL) signaling pathway. We found that in cisplatin-injected mice, there was an increase in the levels of RIPK1, RIPK3, and p-MLKL in the kidneys (Figure 9A,B). Importantly, trametinib treatment markedly reduced the expression of these proteins (RIPK1: 10.51 ± 0.39 versus 1.78 ± 0.18, *p* < 0.001; RIPK3: 12.82 ± 0.63 versus 2.08 ± 0.28, *p* < 0.001; p-MLKL: 2.62 ± 0.31 versus 0.84 ± 0.06, *p* < 0.01; Figure 9A,B), suggesting that trametinib provides protection against necroptosis.

## 3. Discussion

This study explored trametinib’s role in addressing cisplatin-induced AKI, focusing on its effect on MEK and ERK phosphorylation in kidney tissues. Consistent with previous studies [14,15,16], our findings demonstrated increased MEK and ERK phosphorylation in the kidneys following cisplatin exposure, suggesting ERK pathway activation as a response to cisplatin treatment. Notably, trametinib administration post-cisplatin exposure significantly mitigated renal dysfunction, underscoring its therapeutic potential in countering cisplatin-induced renal challenges. A notable observation was trametinib’s effectiveness in reducing tubular injury. Histopathological analysis revealed marked improvements in tubular structures, with increased areas positively stained for LTL and decreased levels of tubular injury markers (KIM-1 and NGAL). LTL is a specific biomarker widely used in histopathological studies for staining the brush border of the proximal tubule in the kidney [21]. This lectin has a selective affinity for particular carbohydrate sequences in the glycoproteins of the brush border, thereby serving as an effective means to visualize this important cellular structure [22]. These results align with trametinib’s known mechanism of action, as an inhibitor of the MAPK/ERK pathway, and its established role in modulating cellular processes critical to renal health [11,12,13,14,15,16]. A recent real-world study has documented cases of AKI associated with the use of MEK inhibitors, including trametinib, and demonstrated their toxicity in human kidney-derived cell lines [23]. Despite these findings, researchers have concluded that AKI related to trametinib is rare and reversible [23]. Therefore, while it is important to continue investigating the nephrotoxic potential of trametinib, the existing evidence does not indicate a significant risk. In our study, we have clearly demonstrated trametinib’s protective effects against cisplatin-induced AKI. This finding is supported by other animal studies, which have also verified the efficacy of trametinib in treating renal diseases [24,25]. Therefore, ongoing attention to the balance between trametinib’s renoprotective effects and potential renal toxicity remains imperative.

While the precise mechanism of cisplatin-induced AKI remains not fully understood, inflammation is widely recognized as a key factor in its pathophysiology [2,3,4]. Cisplatin’s administration is known to trigger an influx of immune cells into the kidneys, exacerbating the production of cytokines and leading to significant renal damage [26,27]. In this study, trametinib effectively curtailed the infiltration of galectin-3-positive macrophages into the kidneys in mice injected with cisplatin. Serum and renal levels of key pro-inflammatory markers such as TNF-α, IL-6, and IL-1β were also reduced, indicating trametinib’s suppressive impact on both systemic and local renal inflammation. The role of TNF-α in the development of cisplatin-evoked AKI is particularly notable. Previous studies have shown that mice lacking TNF-α exhibit resistance to kidney damage from cisplatin [28,29]. Pharmacological inhibition of TNF-α not only reduces the production of various cytokines and chemokines triggered by cisplatin, but also diminishes the severity of renal injury [29]. In our study, following cisplatin administration, there was an induced phosphorylation and degradation of IκBα, leading to increased phosphorylation of the NF-κB p65 subunit. However, these changes were significantly reversed with trametinib treatment, suggesting an inhibitory effect on NF-κB activation. This finding is in line with the established role of the ERK/NF-κB pathway in pro-inflammatory cytokine production and the initiation of inflammation [30,31,32]. For instance, in diabetic nephropathy (DN), enhanced activity of Src homology-2 domain-containing protein tyrosine phosphatase-2 (SHP2), a protein known to activate ERK, has been observed in both human patients and a mouse model [33]. This activation correlates with increased ERK phosphorylation and associated renal inflammation. Inhibiting SHP2 in the mouse model of DN led to decreased ERK/NF-κB activation and subsequent alleviation of renal damage, underlining the critical role of the ERK-NF-κB axis in kidney inflammation [33].

Oxidative stress plays a critical role in the development of cisplatin-induced AKI [2,3,4]. The administration of cisplatin leads to its accumulation in kidney cells, where it instigates the production of ROS [5]. These ROS inflict substantial damage on cellular structures such as lipids, proteins, and DNA within the kidney cells. Such damage triggers a cascade of detrimental effects, including inflammatory responses, mitochondrial dysfunction, activation of cell death pathways, and adverse effects on kidney vasculature, collectively impairing kidney function [2,3,4]. Given the central role of oxidative stress in cisplatin-evoked AKI, managing it emerges as a potential therapeutic strategy [5]. In this study, trametinib significantly suppresses the oxidative stress induced by cisplatin. This is evidenced by the observed reduction in lipid peroxidation by-products (4-HNE and MDA) and the reversal of the GSH/GSSG ratio, a key indicator of oxidative stress, following trametinib treatment. Furthermore, our findings indicate that trametinib effectively downregulates the mRNA and protein expression of NOX4 in mice treated with cisplatin. NOX4, primarily responsible for ROS production in the kidney, plays a vital role in maintaining cellular function and redox balance under normal conditions [34]. However, during cisplatin-induced AKI, NOX4’s role becomes increasingly complex and harmful [34,35]. Previous studies have corroborated our findings, showing an upregulation of NOX4 expression in response to cisplatin, which contributes to oxidative stress [35,36]. This stress, in turn, can activate various inflammatory pathways and lead to fibrotic changes in the kidney, exacerbating initial injury and potentially leading to chronic kidney disease if the injury persists [37,38]. Given its critical role in mediating oxidative stress and subsequent kidney damage, targeting NOX4 has emerged as a promising therapeutic approach [34]. Inhibiting NOX4 activity has the potential to reduce ROS production, thereby mitigating oxidative stress and offering protection against cisplatin-induced kidney damage. Several studies suggest that ERK activation can lead to increased expression of NOX4 [39,40,41]. Consistent with previous research, our study also suggests that ERK activation is associated with an increase in NOX4 expression, further underscoring the potential of trametinib in this context.

Tubular cellular death is a defining feature of AKI induced by cisplatin [2,3,4]. In our research, we observed that trametinib considerably lessened apoptosis in the kidneys of mice treated with cisplatin, as demonstrated by a significant reduction in the number of cells positive for TUNEL staining. The anti-oxidant effect of trametinib can preserve cellular integrity, thereby preventing the cascade of events leading to apoptosis. Moreover, a recent study has highlighted the role of ERK activation in renal injury caused by cisplatin, especially in mitochondrial damage, autophagy, and apoptosis [42]. Inhibition of ERK disrupts mitochondrial damage and subsequent apoptotic signaling pathways induced by cisplatin. This action likely involves the modulation of various cellular processes, including mitochondrial function, autophagy, and the regulation of pro-apoptotic and anti-apoptotic signals [42]. Thus, the observed reduction in apoptosis with trametinib treatment could also result from direct interference in the ERK-mediated apoptotic pathways induced by cisplatin. Beyond apoptosis, necroptosis is increasingly recognized as a significant contributor to cisplatin-evoked AKI [8]. This type of cell death is marked by the interaction and subsequent phosphorylation of RIPK1 and RIPK3, culminating in the activation and membrane translocation of MLKL, leading to membrane disruption. A previous study has shown that mice lacking either RIPK3 or MLKL exhibit a reduced severity of renal damage following cisplatin injection [43]. Additionally, pharmacological suppression of RIPK1 has been shown to lessen both necroptosis in tubular cells and overall renal injury in models of cisplatin administration [44,45]. In our study, we observed that trametinib markedly attenuated cisplatin-evoked necroptosis, evident from the reduced levels of RIPK1, RIPK3, and p-MLKL. These findings indicate that trametinib effectively counteracts two principal mechanisms of tubular cell death in cisplatin-evoked AKI.

While conducting this study, Brown et al. first reported the effects of trametinib on cisplatin-induced nephrotoxicity [20]. According to their findings, although trametinib increased survival rates, it did not improve AKI in a short-term, high-dose model of cisplatin-induced AKI. The discrepancies between our results and those of Brown’s study are challenging to pinpoint, yet they might be cautiously attributed to significant variations in experimental design and methodology. In our study, trametinib was administered at a dose of 3 mg/kg by oral gavage for three days post-cisplatin administration, while Brown et al. administered trametinib at a lower dose of 1 mg/kg by intraperitoneal injection for the same duration. The higher dosage and oral route in our study could have led to different bioavailability and systemic exposure to trametinib, potentially enhancing its efficacy in mitigating renal damage. The route of administration may also affect the pharmacokinetics and pharmacodynamics of trametinib, influencing its interaction with cellular and molecular targets involved in cisplatin-induced renal injury. Moreover, the cisplatin dosage also varied significantly between the two studies, with our study using 20 mg/kg and Brown’s study using a higher dose of 30 mg/kg. The increased dose in Brown’s study likely induced more severe nephrotoxic effects, possibly surpassing the threshold of trametinib’s protective capability against the heightened oxidative stress, inflammation, and cellular damage induced by higher cisplatin levels. Lastly, while Brown’s study was limited to evaluating serum markers such as creatinine and BUN, our study incorporated these assessments alongside detailed histopathological examinations. We also explored a broader range of biomarkers and signal transduction pathways related to inflammation, oxidative stress, tubular cell death. This comprehensive approach not only provided a more detailed characterization of renal damage, but also allowed us to observe the multifaceted protective effects of trametinib. Therefore, the conflicting results between our study and Bowen’s study could be reflective of these fundamental differences in experimental design, suggesting that the efficacy of trametinib in preventing cisplatin-induced AKI may be highly dependent on the specific conditions under which it is tested.

This study has several key limitations that warrant further investigation. First, the use of a mouse model, while valuable for initial investigations, may not fully replicate complex human physiological responses to treatment. Consequently, the findings from this study should be cautiously interpreted when considering translational implications. Furthermore, the absence of larger animal models limits the scope of our conclusions. Including such models in future studies could provide more comprehensive insights and enhance the applicability of our results to clinical settings. Second, we utilized a high-dose, short-term model in this study to evaluate the effect of trametinib on cisplatin-induced AKI. This model is extensively used to rapidly assess the efficacy and mechanisms of potential nephroprotective agents under severe stress scenarios that might occur during clinical emergencies or overdose situations [46]. However, this model does not fully replicate the gradual onset and progression of nephrotoxicity typically observed with the clinical use of cisplatin administered at lower doses over prolonged periods. To address this gap, further research using a low-dose, long-term model is essential to enhance the translational relevance of our findings for clinical applications.

## 4. Materials and Methods

### 4.1. Animals and Treatment

**Figure 10:**
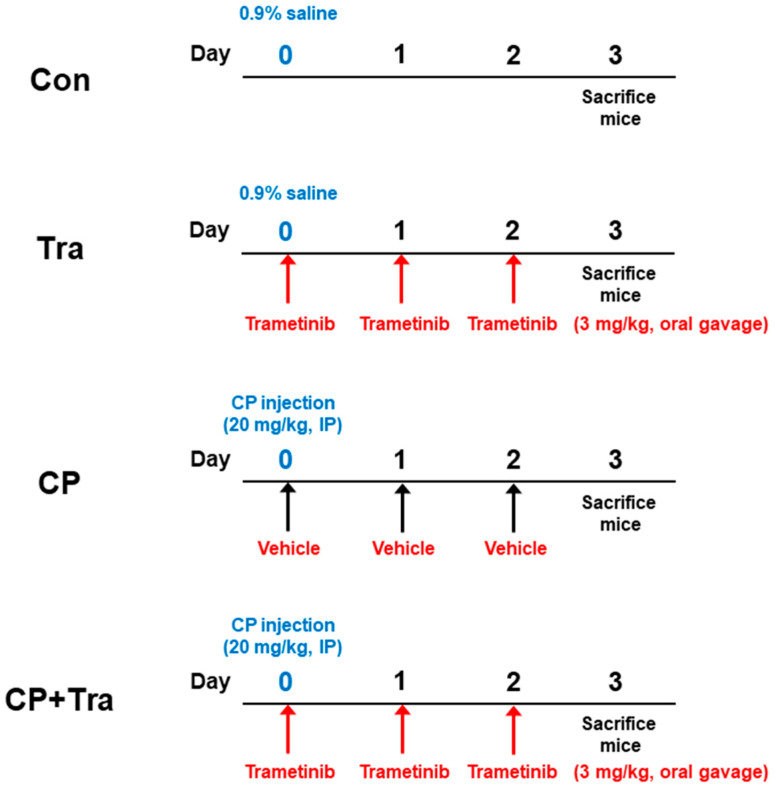
Overview of the animal experiment protocol.

Animal experiments were conducted in accordance with the protocols approved by the Institutional Animal Care and Use Committee of the Daegu Catholic University Medical Center (DCIAFCR-190925-03-Y). Male C57BL/6N mice, 7 weeks old, were obtained from HyoSung Science (Daegu, Korea) and maintained at 20–24 °C with 55% humidity for one week. The mice were randomly divided into four groups (n = 8 per group): control (Con), trametinib (Tra), cisplatin (CP), and a combination of cisplatin and trametinib (CP + Tra). The sample size (n = 8 per group) was determined based on calculations using G*Power software version 3.1.9.4 (Heinrich-Heine-Universität Düsseldorf, Düsseldorf, Germany), with a significance level of 5%, a minimum power of 80%, and a large effect size (0.7). Both the CP and the CP + Tra groups received a single intraperitoneal injection of cisplatin (20 mg/kg, dissolved in 0.9% saline; Sigma-Aldrich, St. Louis, MO, USA) at a volume of 10 μL/g body weight. The Con group received a single intraperitoneal injection of an equal volume of 0.9% saline. The Tra and CP + Tra groups were orally administered trametinib [3 mg/kg body weight per day in 200 μL of 1% carboxymethylcullulose, 0.4% Tween80, and 5% dimethyl sulfoxide (DMSO)] daily for three consecutive days, starting from one hour after the cisplatin injection. The CP group was administered an oral administration of an equal volume of the vehicle for trametinib daily for three consecutive days. All mice were euthanized by intraperitoneal injection of tribromoethanol (500 mg/kg; Sigma-Aldrich) 72 h after a single dose of cisplatin. Blood samples were collected through cardiac puncture, processed to separate serum, and the serum was then stored at −80 °C for further analysis. After the kidneys were rapidly harvested from the mice, the right kidney was immediately frozen at −80 °C, and the left kidney was fixed in 10% formalin. The animal experiment protocol is summarized in Figure 10. Trametinib was purchased from Cayman Chemical (Ann Arbor, MI, USA). The doses of trametinib and cisplatin were selected based on prior studies [25,47].

### 4.2. Biochemical Measurements

Levels of serum creatinine and BUN levels were quantified utilizing assay kits from BioAssay Systems (Cat. No. DICT-500; Hayward, CA, USA) and Thermo Fisher Scientific (Cat. No. EIABUN; Waltham, MA, USA), respectively, adhering strictly to the supplied instructions. The concentrations of pro-inflammatory cytokines TNF-α, IL-6, and IL-1β in serum were determined through ELISA kits provided by R&D Systems (Cat. No. MTA00B for TNF-α, Cat. No. M6000B for IL-6, Cat. No. MLB00C for IL-1β; Minneapolis, MN, USA), following the standard protocols. The renal content of MDA was measured using a kit from Sigma-Aldrich (Cat. No. MAK085), executed per the manufacturer’s guidelines. Additionally, the GSH/GSSG ratio in kidney tissues was evaluated using a kit from Enzo Life Sciences (Cat. No. ADI-900-160; Farmingdale, NY, USA), in accordance with the manufacturer′s recommendations.

### 4.3. Histopathological and Immunostaining Procedures

The formalin-fixed kidney samples were sequentially dehydrated in ethanol, cleared in xylene, and then embedded in paraffin. Thin sections were then prepared and mounted on glass slides. H&E and PAS stains were applied to assess tubular injury. The percentage of damaged tubules, indicated by tubular cell necrosis, tubular dilatation, loss of the brush border, and cast formation, was evaluated across five randomly selected fields at 200× magnification. Tubular injury was scored on a scale from 0 to 5 based on the percentage of damaged tubules: 0 for 0% damage, 1 for ≤10%, 2 for 11–25%, 3 for 26–50%, 4 for 51–75%, and 5 for 76–100%. This scoring was performed in a blinded manner [48]. For IHC, sections were incubated with primary antibodies targeting KIM-1, 4-HNE, and galectin-3 (Cat. No. ab78494 for KIM-1, Cat. No. ab48506 for 4-HNE, Cat. No. ab2785 for galectin-3; all sourced from Abcam, Cambridge, MA, USA), followed by suitable secondary antibodies. To visualize the brush border of proximal tubules, FITC-labeled LTL obtained from Vector Laboratories (Cat. No. FL-1321-2; Burlingame, CA, USA) was applied. Nuclei were counterstained with 4’, 6-diamidino-2-phenylindole (DAPI). Positive staining for KIM-1, 4-HNE, or LTL was quantified using an image analysis software version 11.0 (IMT i-Solution, Coquitlam, BC, Canada) in five arbitrarily chosen fields per sample at 400× magnification. The number of galectin-3-positive cells was counted in five randomly selected fields per sample at 200× magnification. All quantitative analyses were performed in a blinded manner.

### 4.4. Western Blot Analysis

Protein extraction from kidney tissues was performed using a lysis buffer, and proteins were then separated on gradient polyacrylamide gels (Bio-Rad Laboratories, Hercules, CA, USA). Transfer to nitrocellulose membranes was followed by incubation with primary antibodies against various targets, including MEK (1:1000; Cat. No. 8727; Cell Signaling Technology, Danvers, MA, USA), p-MEK (1:1000; Cat. No. 9121; Cell Signaling Technology), ERK (1:1000; Cat. No. 9102; Cell Signaling Technology), p-ERK (1:1000; Cat. No. 9101; Cell Signaling Technology), NGAL (1:1000; Cat. No. sc-515876; Santa Cruz Biotechnology, Santa Cruz, CA, USA), TNF-α (1:1000; Cat. No. ab66579; Abcam), IL-6 (1:1000; Cat. No. ab259341; Abcam), IκBα (1:1000; Cat. No. 4812; Cell Signaling Technology), p-IκBα (1:1000; Cat. No. 2859; Cell Signaling Technology), NF-κB p65 (1:1000; Cat. No. 8242; Cell Signaling Technology), p-NF-κB p65 (1:1000; Cat. No. 3033; Cell Signaling Technology), NOX4 (1:1000; Cat. No. NB110-58849; Novus Biologicals, Littleton, CO, USA), cleaved caspase-3 (1:1000; Cat. No. 9661; Cell Signaling Technology), cleaved PARP-1 (1:1000; Cat. No. 9544; Cell Signaling Technology), p53 (1:1000; Cat. No. 2524; Cell Signaling Technology), Bax (1:1000; Cat. No. sc-7480; Santa Cruz Biotechnology), RIPK1 (1:1000; Cat. No. 4926; Cell Signaling Technology), RIPK3 (1:1000; Cat. No. 95702; Cell Signaling Technology), p-MLKL (1:1000; Cat. No. 37333; Abcam), MLKL (1:1000; Cat. No. 37705; Cell Signaling Technology), and glyceraldehyde-3-phosphate dehydrogenase (GAPDH; 1:3000; Cat. No. 5174; Cell Signaling Technology). GAPDH served as the loading control. Detection was achieved with horseradish peroxidase-linked secondary antibodies and enhanced chemiluminescence reagents (Cat. No. 32109; Thermo Fisher Scientific). Band intensity quantification utilized ImageJ software version 1.53j (National Institutes of Health, Bethesda, MD, USA).

### 4.5. Real-Time Reverse Transcription-Polymerase Chain Reaction (RT-PCR)

RNA extraction from kidney tissues was completed using TRIzol reagent, with reverse transcription conducted using the PrimeScript RT Reagent Kit (Cat. No. RR037A; TaKaRa, Tokyo, Japan) as per the manufacturer’s instructions. Real-time RT-PCR was performed with specific primers (Table 1), employing the Thermal Cycler Dice Real-Time System III (TaKaRa) and Power SYBR Green PCR Master Mix (Cat. No. 4367659; Thermo Fisher Scientific). GAPDH was selected for normalization.

### 4.6. TUNEL Staining

For assessing apoptosis, TUNEL staining was performed using a kit from Roche Diagnostics (Cat. No. 11684795910; Indianapolis, IN, USA), following the guidelines provided by the manufacturer. Nuclei were counterstained with DAPI. Apoptotic cells, identified through TUNEL staining, were quantified in five randomly chosen microscopic fields at a magnification of 600× for each kidney tissue sample.

### 4.7. Statistical Analysis

Data are presented as mean ± standard error of the mean (SEM). Group comparisons were conducted using one-way analysis of variance (ANOVA) complemented by Bonferroni’s post hoc tests to discern statistical differences. Statistical significance was established at *p* values less than 0.05 for all analyses.

## 5. Conclusions

In conclusion, our research provides valuable insights into the potential of trametinib, an FDA-approved oral MEK1/2 inhibitor, as a therapeutic agent against cisplatin-induced AKI. By attenuating key factors like inflammation, oxidative stress, and tubular cell death, trametinib emerges as a promising candidate for enhancing renal protection in cisplatin therapies. These findings not only underscore trametinib’s therapeutic potential, but also pave the way for further clinical exploration of its role in nephrotoxicity mitigation.

## Figures and Tables

**Table 1:** List of primers.

Gene	Primer Sequence (5′ → 3′)	Accession No.
TNF-α	F: CACAGAAAGCATGATCCGCGACGT R: CGGCAGAGAGGAGGTTGACTTTCT	NM_013693
IL-6	F: TAGTCCTTCCTACCCCAATTTCC R: TTGGTCCTTAGCCACTCCTTC	NM_031168
IL-1β	F: CGCAGCAGCACATCAACAAGAGC R: TGTCCTCATCCTGGAAGGTCCACG	NM_008361
NOX4	F: CCCAAGTTCCAAGCTCATTTCC R: TGGTGACAGGTTTGTTGCTCCT	NM_015760
GAPDH	F: ACTCCACTCACGGCAAATTC R: TCTCCATGGTGGTGAAGACA	NM_001289726

## Data Availability

The data presented in this study are available in the article.
